# Supplementary material for: A sequence-aware merger of genomic structural variations at population scale
Source: Nat Commun. 2024 Feb 2;15:960. doi: 10.1038/s41467-024-45244-9 (PMC10837428; doi:10.1038/s41467-024-45244-9)
Supplement: Supplementary file 1 — Supplementary Information [file 41467_2024_45244_MOESM1_ESM.pdf]

A sequence-aware merger of genomic structural variations at population scale

Zheng *et al.*

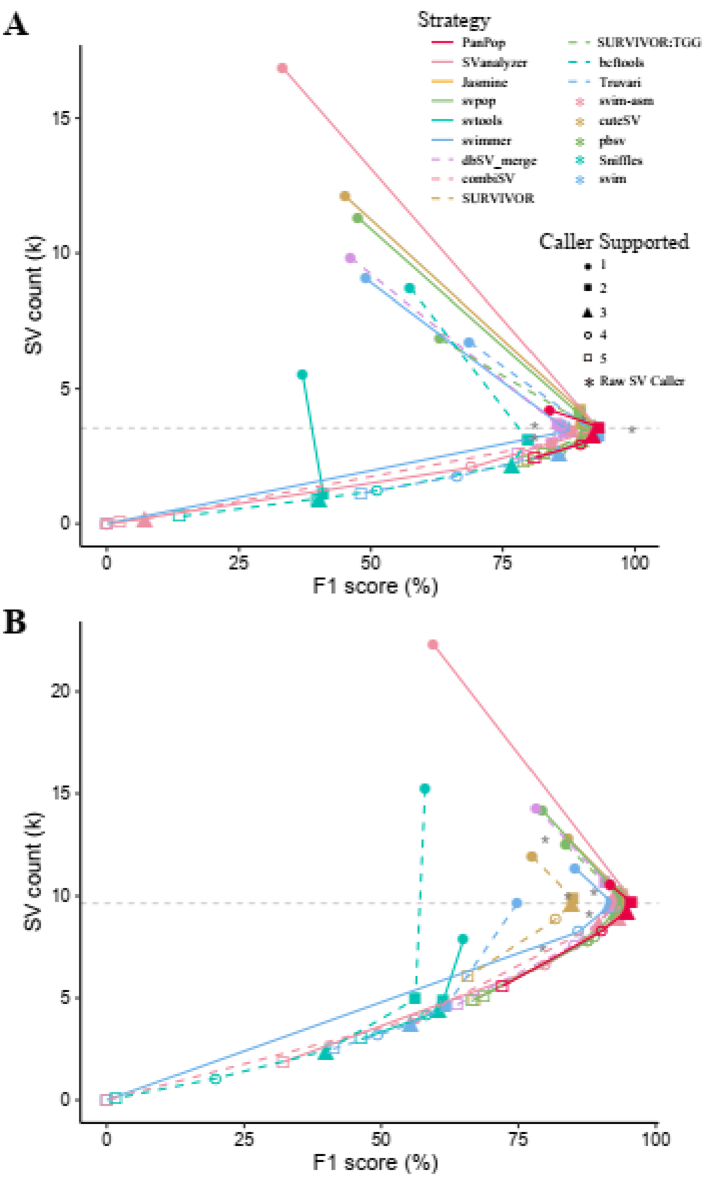

Supplementary Fig. 1. Full diagram comparison of 12 SV mergers and 5 SV callers of single individual SV merging. Results of *A. thaliana* simulated dataset and HG002 dataset were illustrated by **A** and **B**, respectively**.** For each merger, minimal supported SV caller count of 1 to 5 were represented solid circle, solid square, solid triangle, hollow circle and hollow square, and SV callers were marked as asterisk. SV mergers and callers represented using different colors and line types. Source data are provided as a Source Data file.

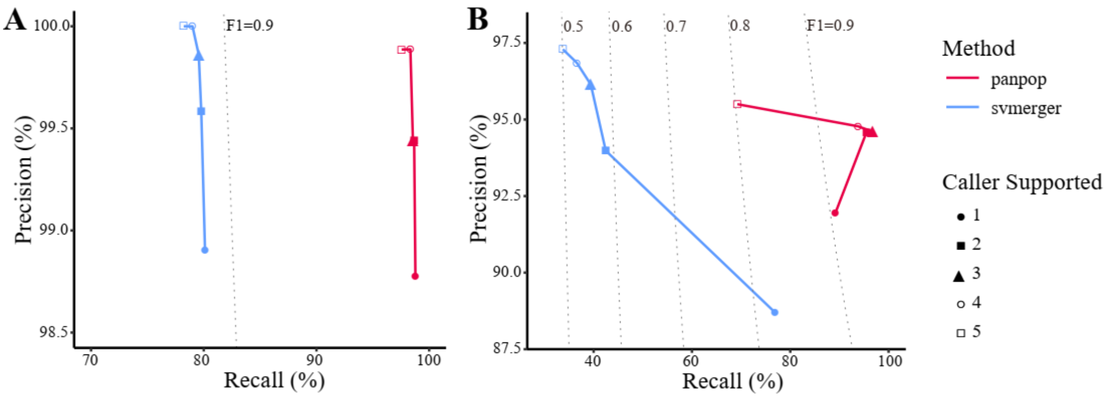

Supplementary Fig. 2. Performance evaluation of sv-merger and PanPop for single individual SV merging. Results of *A. thaliana* simulated dataset and HG002 dataset were illustrated by **A** and **B**, respectively. Red line represented results of PanPop, while the blue line represented results of sv-merger. The performance was assessed by recall (x-axis), precision (y-axis), and F1 score (F1, dashed line). Noted only deletion SVs were included. For each of the two SV mergers, minimal supported SV caller count of 1 to 5 were represented as solid circle, solid square, solid triangle, hollow circle and hollow square, respectively. Source data are provided as a Source Data file.

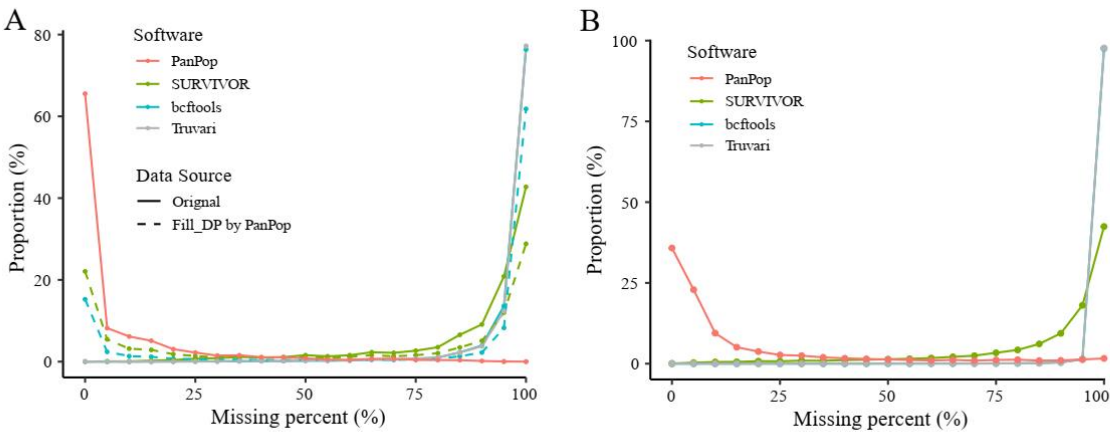

Supplementary Fig. 3. Missing rate of PanPop, SURVIVOR, bcftools and Truvari after merging. **A**, Results of the dataset of 86 TGS samples. SV merging using the original results of SV callers or processed by PanPop's "fill_depth_information" method, represented by the solid or dashed lines, respectively. **B**, Results of the dataset of 1,092 NGS samples. Color of red, green, blue and grey represented different software of PanPop, SURVIVOR, bcftools and Truvari, respectively. Source data are provided as a Source Data file.

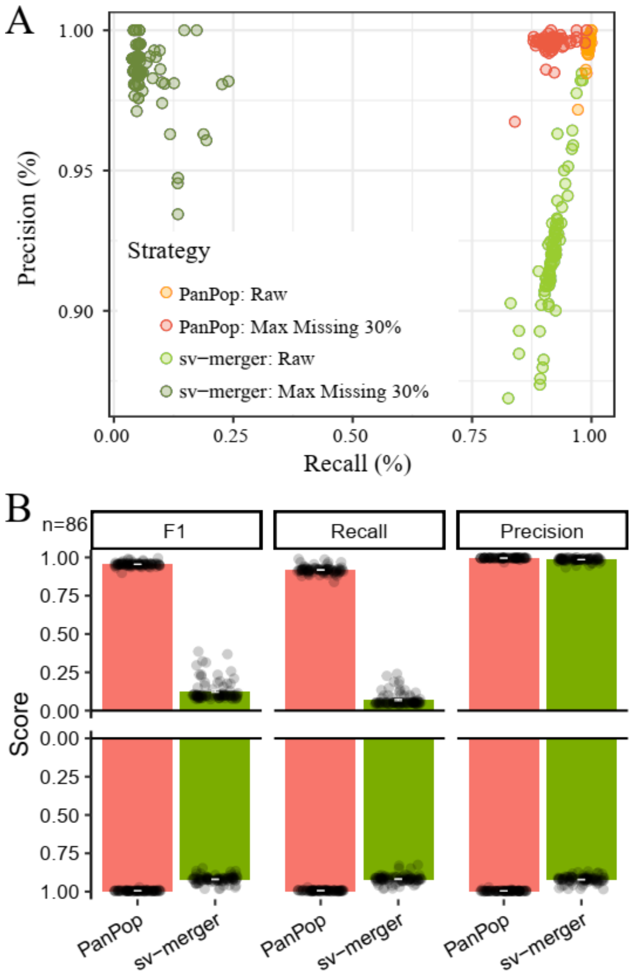

Supplementary Fig. 4. Recall rate, precision rate and F1-score of PanPop and sv-merger using dataset of 86 TGS samples. Noted only deletion SVs were included. Red points and bars represented results of PanPop, while the green points and bars represented results of sv-merger. **A**, the precision and recall rate of PanPop and sv-merger. The bright and dark point represented result of raw SVs and SVs with a filter of max missing of 30%, respectively. **B** shows the histogram of evaluation scores (recall, precision and F1) of the results from PanPop and sv-merger. The upper and lower sections represent the datasets after a maximum missing rate filter of 30% and the raw datasets, respectively. Bars are mean values and white error bars were SE (n=86). Source data are provided as a Source Data file.

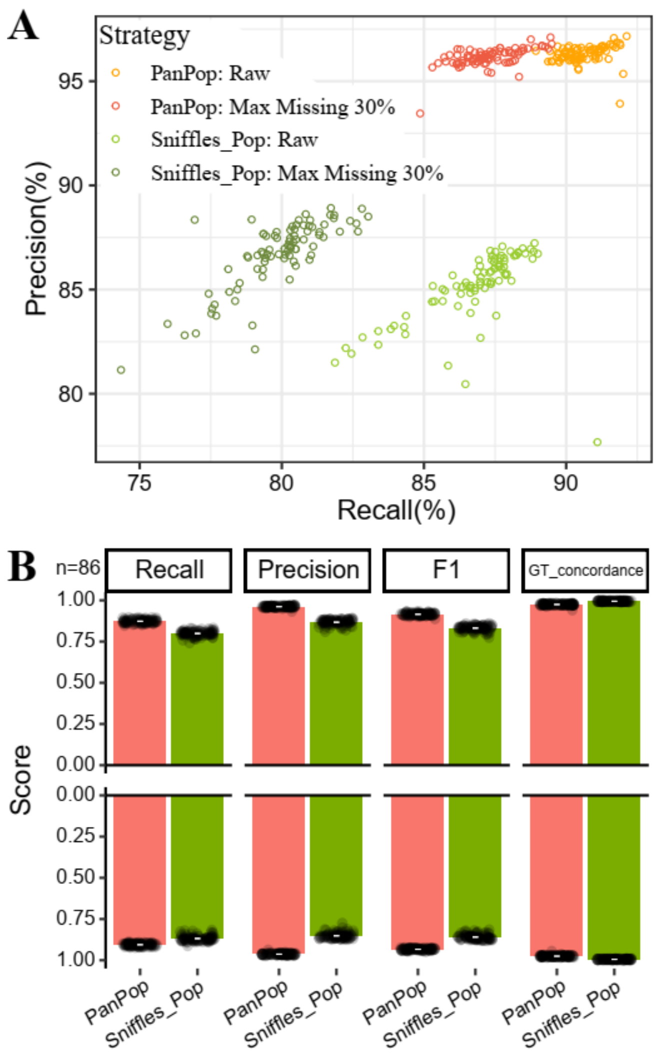

Supplementary Fig. 5. Recall rate, precision rate and F1-score of PanPop and Snffles2. Red points and bars represented results of PanPop, while the green points and bars represented results of Sniffles2. **A**, the precision and recall rate of PanPop and Sniffles2. The bright and dark point represented result of raw SVs and SVs with a filter of max missing of 30%, respectively. **B** shows the histogram of evaluation scores (recall, precision and F1) of the results from PanPop and Sniffles2. The upper and lower sections represent the datasets after a maximum missing rate filter of 30% and the raw datasets, respectively. Bars are mean values and white error bars were SE (n=86). Source data are provided as a Source Data file.

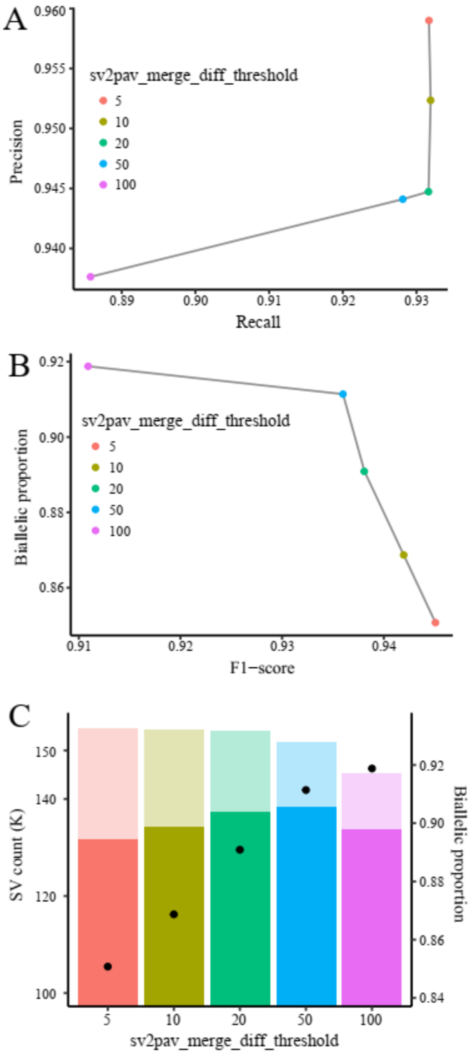

Supplementary Fig. 6. Performance of PanPop using different parameter of group length threshold (sv2pav_merge_diff_threshold) of 5, 10, 20, 50 and 100bp. A, recall and precision score of different sv2pav_merge_diff_threshold parameter. **B**, F1-score and biallelic proportion of different sv2pav_merge_diff_threshold parameter. **C**, biallelic proportion and SV count of different sv2pav_merge_diff_threshold parameter, while the count of all SVs was in shadow bar and count of biallelic SVs was mark as solid bar. The black point shows biallelic proportion.

Supplementary Table 1. Software used for benchmark.

Software	Approach	Version	Website	Citation
SVMergingMethodComparison	-	git@4b4b5a7	https://github.com/mkirsche/SVMergingMethodComparison	[1]
SVIM-asm	SV Caller	v1.0.3	https://github.com/eldariont/svim-asm	[2]
cuteSV	SV Caller	v2.0.2	https://github.com/tjiangHIT/cuteSV	[3]
pbsv	SV Caller	v2.9.0	https://github.com/PacificBiosciences/pbsv	https://github.com/PacificBiosciences/pbsv
Sniffles	SV Caller	v2.0.7	https://github.com/fritzsedlazeck/Sniffles	[4]
svim	SV Caller	v1.4.2	https://github.com/eldariont/svim	[5]
combiSV	pos	v2.2	https://github.com/ndierckx/combiSV	[6]
dbSV_merge	pos	git@85b3687	https://github.com/GrandOmics/svmerge	https://github.com/GrandOmics/svmerge
SURVIVOR*	pos	git@ed1ca51	https://github.com/fritzsedlazeck/SURVIVOR	[7]
SURVIVOR:TGG**	pos	git@291a01a	https://github.com/YaoZhou89/TGG	[8]
svimmer*	pos	git@7fd78b2	https://github.com/DecodeGenetics/svimmer	[9]
svtools*	pos	v0.5.1	https://github.com/hall-lab/svtools	[10]
sv-merger	pos	git@b774523	https://github.com/dbeyter/sv-merger	[11]
**PanPop**	**pos+seq**	**v0.4**	**https://github.com/starskyzheng/panpop**	**This paper**
Jasmine*	pos+seq	v1.1.5	https://github.com/mkirsche/Jasmine	[1]
bcftools	pos+seq	v1.17	https://github.com/samtools/bcftools	[12]
SVanalyzer	pos+seq	v0.36	https://github.com/nhansen/Svanalyzer	[13]
svpop*	pos+seq	v3.4.1	https://github.com/EichlerLab/svpop	[14]
Truvari	Benchmark	v3.0.0	https://github.com/ACEnglish/truvari	[15]

Note: SV mergers were based on the position of reference were marked as “pos” and others took into account the sequence difference were marked as "pos+seq."

*: performed by SVMergingComparision

**: Based on SURVIVOR

Supplementary Table 2. CPU and RAM usage of 13 SV merges for single individual SV merging of *A. thaliana* simulated dataset.

Software	CPUtime/min	Elapsed time/min	CPU usage	Max RAM/Mb
bcftool	0.04	0.04	89%	9.0
Jasmine	0.27	0.30	89%	1098.4
SURVIVOR	0.14	0.15	95%	85.2
svimmer	0.03	0.03	92%	98.3
svtools	1.76	1.59	122%	94.6
svpop	13.30	2.22	600%	157.9
dbSV_merge	0.01	0.02	41%	28.7
SVanalyzer	2.11	11.33	19%	46.9
combiSV	0.13	0.13	100%	53.8
TTG	0.23	0.23	99%	196.8
sv-merger	0.08	0.08	93%	51.2
Truvari	0.26	0.19	136%	89.1
PanPop	35.45	3.33	1064%	1724.7

Supplementary Table 3. CPU and RAM usage of four SV merges for population SV merging of *A. thaliana* TGS dataset.

Software	CPU time (min)	Elapsed time (min)	CPU usage	Max RAM (Gb)	Dataset	Data preprocess	Note
bcftool	2.19	1.99	110%	0.29	86 TGS samples	-	
Truvari	9.39	8.39	112%	0.60	86 TGS samples	-	
SURVIVOR	1.21	1.70	71%	0.03	86 TGS samples	-	
sv-merger	3.88	3.94	99%	0.43	86 TGS samples	Deletion-only	
PanPop	2731.44	122.58	2228%	17.35	86 TGS samples	-	
bcftool	2.9145	3.496166667	83%	0.12	86 TGS samples	Fill-Depth-Information	
Truvari	13.93483333	14.13	99%	0.65	86 TGS samples	Fill-Depth-Information	
SURVIVOR	2.926166667	3.328833333	88%	0.50	86 TGS samples	Fill-Depth-Information	
bcftool	323.111	218.5	148%	10.74	1092 NGS samples	-	
Truvari	1264.6495	1394.283333	91%	11.86	1092 NGS samples	-	
SURVIVOR	58.61666667	65.46666667	90%	0.73	1092 NGS samples	-	
PanPop	140396.28	2909	4826%	57.89	1092 NGS samples	-	5 nodes were used
bcftool	2250.296	1471.916667	153%	11.05	1092 NGS samples	Fill-Depth-Information	
Truvari	NA	More than 7 days	Single thread	1.44*	1092 NGS samples	Fill-Depth-Information	
SURVIVOR	NA	More than 7 days	Single thread	33.59*	1092 NGS samples	Fill-Depth-Information	

* It has been running for more than 7 days and still incomplete. The RAM usage was the maximal during this time.

Supplementary Table 4. PanPop performance of Repeat and non-repeat regions.

Chi-Squared test (df=1) was used.

Region	***A. thaliana* simulated dataset**		**Real HG002 dataset**	
	**Repeat**	**Non-repeat**	**Repeat**	**Non-repeat**
Base SV count	520	2717	4585	2859
Call SV count	513	2591	4533	2844
FP SV count	17	66	147	104
FN SV count	24	192	199	119
precision	96.69%	97.45%	96.76%	96.34%
recall	95.38%	92.93%	95.66%	95.84%
F1	96.03%	95.14%	96.21%	96.09%
FP *p*-value	0.3532		0.3662	
FN *p*-value	0.066		0.768	

Supplementary Table 5. Software used by PanPop.

Software	Function type	Version	Website	Citation
Assemblytics	SV Caller	v1.2.1	https://github.com/MariaNattestad/Assemblytics	[16]
cuteSV	SV Caller	v2.0.2	https://github.com/tjiangHIT/cuteSV	[3]
pbsv	SV Caller	v2.9.0	https://github.com/PacificBiosciences/pbsv	https://github.com/PacificBiosciences/pbsv
Sniffles	SV Caller	v2.0.7	https://github.com/fritzsedlazeck/Sniffles	[4]
svim	SV Caller	v1.4.2	https://github.com/eldariont/svim	[5]
SVIM-asm	SV Caller	v1.0.3	https://github.com/eldariont/svim-asm	[2]
MUSCLE	Sequence Aligner	v3.8.31	https://drive5.com/muscle/downloads_v3.htm	[17]
MUMmer	Sequence Aligner	v3.23	https://github.com/mummer4/mummer	[18]
FAMSA	Sequence Aligner	v2.2.2	https://github.com/refresh-bio/FAMSA	[19]
stmsa	Sequence Aligner	v0.2.1	https://github.com/malabz/stmsa-cpp	[20]
mimimap2	Reads Mapper	v2.24	https://lh3.github.io/minimap2	[21]
NGLMR	Reads Mapper	v0.2.7	https://github.com/philres/ngmlr	[4]
VG	Reads Mapper & SV Caller	v1.36.0	https://github.com/vgteam/vg	[22]
bcftools	Raw SV merger	v1.17	https://github.com/samtools/bcftools	[12]
samtools	Bam sorter & depth calculator	v1.17	https://github.com/samtools/samtools	[12]
bedtools	Bed merger	v2.23.0	https://bedtools.readthedocs.io/en/latest/	[23]
Minigraph	Graph genome builder	v0.20	https://lh3.github.io/minigraph/	[24]

Supplementary Note 1. Benchmark for sv-merger

The example and test dataset using deletion only on the GitHub website (https://github.com/dbeyter/sv-merger). Hence, only deletion was used to valuated performance of sv-merge. As for PanPop, the deletion-only SVs were used to performance. Benchmark for sv-merger and PanPop were also based on deletion-only SV dataset.

Supplementary Note 2. Benchmark for Sniffles2 for population SV

Firstly, we perform SV calling using Sniffles2 for each samples, which generate '.VCF' and '.snf' for each individual. Then, Sniffles2 and PanPop were used to combination a VCF of population SV, separately. Benchmark for Sniffles2 and PanPop were also based on SVs called by Sniffles2 of single samples.

Supplementary Note 3. Runtime and resource consuming

All the computing were based on a cluster of Intel Xeon Gold 6248 CPU @ 2.50GHz*2 with 192G of memory. PanPop were designed for parallel running, not only available for single node, but also support running over multiple nodes of a cluster. Here we used one node for single individual SV calling and merging, and the merging process of TGS dataset of A. thaliana. Since the dataset of 1,092 NGS were much more complex, five of those nodes were used for running.

PanPop used Multiple sequence alignment methods, which are typically more computationally intense. Performance of PanPop consuming more computational resource, as a high precision in return. But PanPop were designed for parallel running even cross multiple nodes. As for single individual SV merging, The CPU time of PanPop were about 15-times higher than SVanalyzer and about 248-times higher than

SURVIVOR (Supplementary Table 3). Benefited by the optimize of parallel computing, the real elapsed time of PanPop were only 11-times higher than SURVIVOR and 0.3-fold of SVanalyzer. The memory usage of PanPop were 1.7Gb, which is higher than other software, but if very tolerable even for a laptop.

As for population SV merging of TGS dataset, there were a process of 'fill-depth-information' which reduce the speed of PanPop Although 45.5 hours of CPU hour were used for variant merging of PanPop, which is more than two thousand-fold of bcftools and SURVIVOR (Supplementary Table 3). The real time elapsed of about 2-hour for PanPop, which is about 37-fold of SURVIVOR, 35-fold of bcftools and 8.7-fold of Truvari (compared with the data preprocess of Fill-Depth-Information).

As for population SV merging of NGS dataset, the performance is similarly with TGS dataset. Although 97.5 days of CPU hour were used for variant merging of PanPop. But since PanPop support parallel over multiple nodes, as for using 5 nodes for computing, only 48 hours were needed (Supplementary Table 3). As a compare, by using a dataset of preprocess of Fill-Depth-Information, SURVIVOR and Truvari not complete after 7 days of running, The bcftools failed to perform `norm` function, which is the important process of SV merging of bcftools pipeline. Still, the partly result of Truvari and bcftools shows a highly missing rate and partly overlapped SVs cannot be process properly.

Supplementary Note 4. Simulating structure variants

We use VISOR to simulated genome-wide SVs. The telomere and centromeres regions were excluded which were annotated by Hou[25].

Firstly, we simulated 4 events of genome wide SVs with VISOR

Rscript randomregion.r -d /path/to/genome.fasta.dim -n SV_number_to_simulate -l 750 -s 150 -v ' insertion,deletion,tandem duplication,dispersed duplication' -r

‘ 25:25:25:25’ -x /path/to/exclude.bed > /path/to/output.bed

Then, we generated a sequence of haplotype in FASTA format.

VISOR HACk -g /path/to/reference.fa -b /path/to/output.bed -o /path/to/output_hap

Finally, this haplotype was used to simulate HiFi reads.

Awk ‘OFS=FS=”\t””{print $1, “1”, $2, “100.0”, “100.0”}’ /path/to/genome.fasta.dim > /path/to/genome.fasta.dim.shorts.laser.simple.bed

VISOR LASeR -g /path/to/reference.fa -s /path/to/output_hap -b /path/to/genome.fasta.dim.shorts.laser.simple.bed -o /path/to/output_seq –threads 10 –coverage 40 –tag –fastq –compress –glitches_size 20 –glitches_skip 20 –identity_min 95 –identity_max 99 –identity_stdev 2

Supplementary Note 5. SV merging of repeat and non-repeat regions

SVs located in repeat regions can exhibit inconsistent locations and variable SV lengths, which can pose challenges in the merging process. Within PanPop, incorporated two modules could address these issues. The "Realign-group" module gathers nearby SVs together, effectively handling the problem of inconsistent locations. Additionally, the "Local realign" module removes redundant sequences shared by SVs, enabling the extraction of multiple simple SVs from a larger and more complex SV. As a result, PanPop is capable of effectively handling these complex conditions.

Furthermore, we conducted tests to evaluate the performance of PanPop with SVs that are entirely within repeat regions compared to those in non-repeat regions. After performing a chi-square test, we found no significant difference in the false positive (FP) or false negative (FN) counts between the two sets of SVs (Supplementary Table 4).

Supplementary Note 6. Group length threshold parameter

This parameter can be modified through the config file of 'sv2pav_merge_diff_threshold'. 'sv2pav_merge_diff_threshold' influenced the thin process that the two allele should be merged if their sequence difference is smaller than 'sv2pav_merge_diff_threshold'. With the sv2pan_merge_diff_threshold increased, the accuracy decreased (recall, precision and F1 score decreased), and the biallelic proportion increased (Supplementary Fig. 6). Users could set this parameter depends on their certain purpose. In this paper and the default parameter, we set sv2pan_merge_diff_threshold to a compromise value of 20bp.

Supplementary Note 7. Software version and parameters

Minimap2 (v2.24)

For Oxford Nanopore reads:

minimap2 -ax map-ont –MD -Y /path/to/reference.fa /path/to/reads.fq.gz | samtools view –output-fmt BAM -h -o /path/to/output.bam

For PacBio reads:

minimap2 -ax map-pb –MD -Y /path/to/reference.fa /path/to/reads.fq.gz | samtools view –output-fmt BAM -h -o /path/to/output.bam

For HiFi reads:

minimap2 -ax map-hifi –MD -Y /path/to/reference.fa /path/to/reads.fq.gz | samtools view --output-fmt BAM -h -o /path/to/output.bam

Sniffles (v2.0.7)

For single individual SV calling:

sniffles --input /path/to/sample.sorted.bam --vcf /path/to/sniffles.vcf

For population SV calling:

sniffles --input /path/to/sample.sorted.bam --snf /path/to/1/sniffles.snf --threads 20

sniffles --input /path/to/1/sniffles.snf --vcf /path/to/2.sniffles.snf

svim (v1.4.2)

svim alignment /path/to/output /path/to/sample.sorted.bam /path/to/reference.fa --insertion_sequences

cuteSV (v2.0.2)

For Oxford Nanopore reads:

cuteSV /path/to/input.bam /path/to/reference.fa /path/to/output.vcf /path/to/work_dir –genotype --min_read_len 500 --max_cluster_bias_INS 100 --diff_ratio_merging_INS 0.3 --max_cluster_bias_DEL 100 --diff_ratio_merging_DEL 0.3

For PacBio reads:

cuteSV /path/to/input.bam /path/to/reference.fa /path/to/output.vcf /path/to/work_dir –genotype --min_read_len 500 --max_cluster_bias_INS 100 --diff_ratio_merging_INS 0.3 --max_cluster_bias_DEL 200 --diff_ratio_merging_DEL 0.5

For HiFi reads:

cuteSV /path/to/input.bam /path/to/reference.fa /path/to/output.vcf /path/to/work_dir –genotype --min_read_len 500 --max_cluster_bias_INS 1000 --diff_ratio_merging_INS 0.9 --max_cluster_bias_DEL 1000 --diff_ratio_merging_DEL 0.5

pbsv (v2.9.0)

pbsv discover /path/to/input.bam /path/to/output.discover.svsig.gz

pbsv call /path/to/reference.fa /path/to/output.vcf

minimap2 (v2.24)

minimap2 -a -x asm5 --cs -r2k -t 32 /path/to/reference.fa /path/to/queue.fa *2>>{log} | samtools view -b -o /path/to/minimap_genome.bam -*

SVIM-asm (v1.0.3)

svim-asm haploid /path/to/output/dir /path/to/minimap_genome.sorted.bam */path/to/reference.fa*

Truvari (v3.0.0)

For HG002 dataset:

zcat /path/to/HG002_SVs_Tier1_v0.6.vcf.gz | perl -lne 'print if /^#/ or /\tPASS\t/' | bgzip -c > /path/to/HG002_SVs_Tier1_v0.6.PASS.vcf.gz

truvari bench -b /path/to/HG002_SVs_Tier1_v0.6.PASS.vcf.gz -c /path/to/comparisosion-set.vcf.gz -f /path/to/reference.fa --pctsim 0. 5 --pctsize 0.5 –multimatch -o /path/to/output_prefix –includebed /path/to/HG002_SVs_Tier1_v0.6.bed

For other dataset:

truvari bench -b /path/to/truth-set.vcf.gz -c /path/to/comparisosion-set.vcf.gz -f /path/to/reference.fa --pctsim 0. 5 --pctsize 0.5 –multimatch -o /path/to/output_prefix

Besides of PanPop, we used 9 different strategies for mutation merging, which were Jasmine, SVanalyzer, svpop, svtools, svimmer, dbSV merge, combiSV, SURVIVOR and TGG pipeline. Noted that TGG pipeline were mainly based on SURVIVOR.

combiSV (v2.2)

Only four SV caller based on reads were used, because of combiSV not support Assemblytics/SVIM-asm

perl combiSV2.2.pl -pbsv /path/to/pbsv.vcf -cutesv /path/to/cuteSV.vcf -svim /path/to/svim.vcf -sniffles /path/to/sniffles.vcf -o /path/to/output.combiSV.vcf

dbSV_merge (github commit: 85b3687)

ls /path/to/pbsv.vcf /path/to/cuteSV.vcf /path/to/svim.vcf /path/to/sniffles.vcf /path/to/ Assemblytics.vcf > 1.raw.vcf.list

dbSV_merge -f 1.raw.vcf.list -l 2.0 -r 0.4 -o 2.output.tab

SVanalyser (v0.36)

ls /path/to/pbsv.vcf /path/to/cuteSV.vcf /path/to/svim.vcf /path/to/sniffles.vcf /path/to/ Assemblytics.vcf > 1.raw.vcf.list

svanalyzer merge --ref /path/to/reference.fa --fof 1.raw.vcf.list

SVMergingMethodComparison (github commit: 4b4b5a7)

The performance of svtools, svimmer, jasminintra, svpop and SURVIVOR were performed by SVMergingComparision with default parameters.

For jasmine, SURVIVOR, svimmer, svtools:

ls /path/to/pbsv.vcf /path/to/cuteSV.vcf /path/to/svim.vcf /path/to/sniffles.vcf /path/to/ Assemblytics.vcf > 1.raw.vcf.list

/path/SVMergingMethodComparison/runmerging.sh 1.raw.vcf.list 2.output_dir 0

For svpop:

ls /path/to/pbsv.vcf /path/to/cuteSV.vcf /path/to/svim.vcf /path/to/sniffles.vcf /path/to/ Assemblytics.vcf > 1.raw.vcf.list

/path/to/SVMergingMethodComparison/run_svpop.sh 1.raw.vcf.list 2.output_dir 0 /path/to/work_dir

TGG (github commit: 291a01a)

The TGG pipeline were using SURVIVOR as SV merger. Detail steps were described in TGG github pages (https://github.com/YaoZhou89/TGG/tree/main/4.Graph_pangenome/1.construction_g

raph_genome).

sv-merger (github commit: b774523)

Firstly, we extract all SVs from VCFs and convert to a specific csv file by using our perl script:

perl vcf2sv_merger_tsv.pl 2.tsv 1.vcfs_dir/.vcf.gz*

Then, the deletions were extracted:

cat 2.tsv | grep DEL > 2.tsv.del

Then, performance sv-merger:

python2 /path/to/sv-merger/main.py MERGE 2.tsv.del Araport11.rep.merge.bed.gor DEL

Finally, recover VCF from output of sv-merger:

perl result2vcf.pl 2.tsv.del 2.tsv.del.intrr.merged.csv 2.tsv.del.outtrr.merged.csv x1.extract_del.pl.out 3.out.vcf

Supplementary Note 8. SV parse of raw SVs from SV callers

Since not all VCF files from SV calling tools contain sequence information directly. All the necessary information for sequence reconstruction was obtained from the VCF files, which were preprocessed before use. For SV calls from long reads mapping callers, we utilized our script 'long_caller_parser.pl' to effectively extract and organize the relevant information from the Info column of the raw VCF files. This information includes the position of the SV types, the insertion sequences for insertions, and the SV lengths for deletions. With this extracted information, we were able to accurately reconstruct the sequences associated with the SVs. In the case of assembly-based SV callers, the insertion sequence was determined based on the position of the query sequence. By utilizing this positional information, we successfully reconstructed the

sequences related to the SVs.

Supplementary reference

1. Kirsche, M. *et al.* Jasmine and Iris: population-scale structural variant comparison and analysis. *Nat Methods* **20**, 408–417 (2023).

2. Heller, D. & Vingron, M. SVIM-asm: structural variant detection from haploid and diploid genome assemblies. *Bioinformatics* **36**, 5519–5521 (2021).

3. Jiang, T. *et al.* Long-read-based human genomic structural variation detection with cuteSV. *Genome Biology* **21**, 189 (2020).

4. Sedlazeck, F. J. *et al.* Accurate detection of complex structural variations using single-molecule sequencing. *Nat Methods* **15**, 461–468 (2018).

5. Heller, D. & Vingron, M. SVIM: structural variant identification using mapped long reads. *Bioinformatics* **35**, 2907–2915 (2019).

6. Dierckxsens, N., Li, T., Vermeesch, J. R. & Xie, Z. A benchmark of structural variation detection by long reads through a realistic simulated model. *Genome Biology* **22**, 342 (2021).

7. Jeffares, D. C. *et al.* Transient structural variations have strong effects on quantitative traits and reproductive isolation in fission yeast. *Nat Commun* **8**, 14061 (2017).

8. Zhou, Y. *et al.* Graph pangenome captures missing heritability and empowers tomato breeding. *Nature* **606**, 527–534 (2022).

9. Kristmundsdottir, S. *et al.* GraphTyper2 enables population-scale genotyping of structural variation using pangenome graphs. *Nat Commun* **10**, 5402 (2019).

10. Larson, D. *et al.* hall-lab/svtools: svtools v0.5.1. (2019) doi:10.5281/zenodo.3406745.

11. Beyter, D. *et al.* Long-read sequencing of 3,622 Icelanders provides insight into the role of structural variants in human diseases and other traits. *Nat Genet* **53**, 779–786 (2021).

12. Danecek, P. *et al.* Twelve years of SAMtools and BCFtools. *GigaScience* **10**, giab008 (2021).

13. Zook, J. M. *et al.* A robust benchmark for detection of germline large deletions and insertions. *Nat Biotechnol* **38**, 1347–1355 (2020).

14. Ebert, P. *et al.* Haplotype-resolved diverse human genomes and integrated analysis of structural variation. *Science* **372**, eabf7117 (2021).

15. English, A. C., Menon, V. K., Gibbs, R. A., Metcalf, G. A. & Sedlazeck, F. J. Truvari: refined structural variant comparison preserves allelic diversity. *Genome Biology* **23**, 271 (2022).

16. Nattestad, M. & Schatz, M. C. Assemblytics: a web analytics tool for the detection of variants from an assembly. *Bioinformatics* **32**, 3021–3023 (2016).

17. Edgar, R. C. MUSCLE: multiple sequence alignment with high accuracy and high throughput. *Nucleic Acids Research* **32**, 1792–1797 (2004).

18. Kurtz, S. *et al.* Versatile and open software for comparing large genomes. *Genome Biology* **5**, R12 (2004).

19. Deorowicz, S., Debudaj-Grabysz, A. & Gudyś, A. FAMSA: fast and accurate multiple sequence alignment of huge protein families. *Sci Rep* **6**, 33964 (2016).

20. Zou, Q., Hu, Q., Guo, M. & Wang, G. HAlign: fast multiple similar DNA/RNA sequence alignment based on the centre star strategy. *Bioinformatics* **31**, 2475–2481 (2015).

21. Li, H. Minimap2: pairwise alignment for nucleotide sequences. *Bioinformatics* **34**, 3094–3100 (2018).

22. Hickey, G. *et al.* Genotyping structural variants in pangenome graphs using the vg toolkit. *Genome Biology* **21**, 35 (2020).

23. Quinlan, A. R. & Hall, I. M. BEDTools: a flexible suite of utilities for comparing genomic features. *Bioinformatics* **26**, 841–842 (2010).

24. Li, H., Feng, X. & Chu, C. The design and construction of reference pangenome graphs with minigraph. *Genome Biology* **21**, 265 (2020).

25. Hou, X., Wang, D., Cheng, Z., Wang, Y. & Jiao, Y. A near-complete assembly of an *Arabidopsis thaliana* genome. *Molecular Plant* **15**, 1247–1250 (2022).
