# Supplementary material for: A sequence-aware merger of genomic structural variations at population scale
Source: Nat Commun. 2024 Feb 2;15:960. doi: 10.1038/s41467-024-45244-9 (PMC10837428; doi:10.1038/s41467-024-45244-9)
Supplement: Supplementary file 3 — Description of Additional Supplementary Files [file 41467_2024_45244_MOESM3_ESM.pdf]

Description of Additional Supplementary Files

File Name: Supplementary Data 1
Description: Benchmark of SV merging for single individual using simulated dataset.

File Name: Supplementary Data 2
Description: Benchmark of SV merging for single individual using HG002 dataset.

File Name: Supplementary Data 3
Description: Data source of 86 long sequenced *A. thaliana* and the Col-PEK reference.

File Name: Supplementary Data 4
Description: Data source of short sequenced *A. thaliana*.
