# Supplementary material for: A sequence-aware merger of genomic structural variations at population scale
Source: Nat Commun. 2024 Feb 2;15:960. doi: 10.1038/s41467-024-45244-9 (PMC10837428; doi:10.1038/s41467-024-45244-9)
Supplement: Supplementary file 8 — Reporting Summary [file 41467_2024_45244_MOESM8_ESM.pdf]

nature portfolio

Corresponding author(s): Yongzhi Yang, Jianquan Liu

Last updated by author(s): Jan 3, 2024

Reporting Summary

Nature Portfolio wishes to improve the reproducibility of the work that we publish. This form provides structure for consistency and transparency in reporting. For further information on Nature Portfolio policies, see our Editorial Policies and the Editorial Policy Checklist.

Statistics

For all statistical analyses, confirm that the following items are present in the figure legend, table legend, main text, or Methods section.

n/a	Confirmed	
☐	☒	The exact sample size (*n*) for each experimental group/condition, given as a discrete number and unit of measurement
☒	☐	A statement on whether measurements were taken from distinct samples or whether the same sample was measured repeatedly
☐	☒	The statistical test(s) used AND whether they are one- or two-sided<br>*Only common tests should be described solely by name; describe more complex techniques in the Methods section.*
☒	☐	A description of all covariates tested
☒	☐	A description of any assumptions or corrections, such as tests of normality and adjustment for multiple comparisons
☐	☒	A full description of the statistical parameters including central tendency (e.g. means) or other basic estimates (e.g. regression coefficient) AND variation (e.g. standard deviation) or associated estimates of uncertainty (e.g. confidence intervals)
☐	☒	For null hypothesis testing, the test statistic (e.g. *F*, *t*, *r*) with confidence intervals, effect sizes, degrees of freedom and *P* value noted<br>*Give P values as exact values whenever suitable.*
☒	☐	For Bayesian analysis, information on the choice of priors and Markov chain Monte Carlo settings
☒	☐	For hierarchical and complex designs, identification of the appropriate level for tests and full reporting of outcomes
☒	☐	Estimates of effect sizes (e.g. Cohen's *d*, Pearson's *r*), indicating how they were calculated

Our web collection on statistics for biologists contains articles on many of the points above.

Software and code

Policy information about availability of computer code

Data collection	All data were downloaded from NCBI and CNCB databases. The only software used in this process is SRA-toolkit (v3.0.2) which is public in NCBI.
Data analysis	We used many software in our pipeline or during comparison: SVMergingMethodComparison(git@4b4b5a7) Assemblytics(v1.2.1) cuteSV (v2.0.2) pbsv(v2.9.0) Sniffles(v2.0.7) svim(v1.4.2) combiSV(v2.2) dbSV_merge(git@85b3687) SURVIVOR(git@ed1ca51) SURVIVOR:TGG (git@291a01a) svimmer(git@7fd78b2) svtools(git@6a6a7b0) PanPop(v0.3) Jasmine(git@a52773c) SVanalyzer(v0.36) svpop(git@01b26ec) Truvari (v3.0.0) MUSCLE(v3.8.31) MUMmer(v3.23) FAMSA(v2.2.2) stmsa(v0.2.1) mimimap2(v2.24) NGLMR(v0.2.7) VG(v1.36.0) bcftools(v1.17) samtools(v1.17) bedtools(v2.23.0) Minigraph(v0.20). PanPop were available at https://github.com/starskyzheng/panpop.<br>Most of the codes of our pipeline were written by me and is public accsess in GitHub under MIT License. Still, we used source code of MCL algorithm (http://www.micans.org/mcl/scripts/minimcl) which is under GNU General Public License.

For manuscripts utilizing custom algorithms or software that are central to the research but not yet described in published literature, software must be made available to editors and reviewers. We strongly encourage code deposition in a community repository (e.g. GitHub). See the Nature Portfolio guidelines for submitting code & software for further information.

Data

Policy information about availability of data

All manuscripts must include a data availability statement. This statement should provide the following information, where applicable:

- Accession codes, unique identifiers, or web links for publicly available datasets
- A description of any restrictions on data availability
- For clinical datasets or third party data, please ensure that the statement adheres to our policy

The HiFi sequencing data for HG002 are available at GIAB FTP site (https://ftp.ncbi.nlm.nih.gov/ReferenceSamples/giab/data/AshkenazimTrio/HG002_NA24385_son/PacBio_SequelII_CCS_11kb). Raw NGS datasets of Arabidopsis thaliana are available under BioProject accession PRJNA273563. Raw TGS datasets of A. thaliana are available under BioProject PRJCA012695, PRJEB55353, PRJNA715329 and PRJNA834751. The dataset of single individual SV merging in the A. thaliana simulated dataset can be accessed at 10.6084/m9.figshare.24657801, while the HG002 dataset is available at 10.6084/m9.figshare.24658671. Population-scale SV merging results and raw SV data for each TGS sample can be found at 10.6084/m9.figshare.24659160.

Research involving human participants, their data, or biological material

Policy information about studies with human participants or human data. See also policy information about sex, gender (identity/presentation), and sexual orientation and race, ethnicity and racism.

Reporting on sex and gender	Since we only use one public available, human dataset (HG002) for validation. This item is not applicable to this work.
Reporting on race, ethnicity, or other socially relevant groupings	Since we only use one public available, human dataset (HG002) for validation. This item is not applicable to this work.
Population characteristics	Not Applicable
Recruitment	Since we only use one public available, human dataset (HG002) for validation, This item is not applicable to this work.
Ethics oversight	Since we only use one public available, human dataset (HG002) for validation, This item is not applicable to this work.

Note that full information on the approval of the study protocol must also be provided in the manuscript.

Field-specific reporting

Please select the one below that is the best fit for your research. If you are not sure, read the appropriate sections before making your selection.

☒ Life sciences ☐ Behavioural & social sciences ☐ Ecological, evolutionary & environmental sciences

For a reference copy of the document with all sections, see nature.com/documents/nr-reporting-summary-flat.pdf

Life sciences study design

All studies must disclose on these points even when the disclosure is negative.

Sample size	We used 86 TGS samples and 1092 NGS samples of Arabidopsis. We also used TGS dataset of one human (HG002) for validation. We used all public available TGS samples (86 samples) of Arabidopsis., which is greater than most of the sample size of current researches. Considering the anticipated increase in sequencing data volume in the future, a large number of samples will be analyzed. Therefore, we also used 1,092 short-sequenced samples as a replacement to evaluate large-scale population SV merging.
Data exclusions	For samples of short reads of Arabidopsis 1001 Genomes Project, we exclude 43 samples due to short read length or reference itself.
Replication	All dataset were public available, and software and parameters were shown in supplement information. We also provides an example of PART which is available at CodeOcean (https://doi.org/10.24433/CO.1577027.v1).
Randomization	We used all public available TGS dataset and 1092 NGS samples. The result shows a constant trend between samples. Hence, randomization is not applicable to our study.
Blinding	We used all public available TGS dataset and 1092 NGS samples. The result shows a constant trend between samples. Hence, blinding is not applicable to our study.

Reporting for specific materials, systems and methods

We require information from authors about some types of materials, experimental systems and methods used in many studies. Here, indicate whether each material, system or method listed is relevant to your study. If you are not sure if a list item applies to your research, read the appropriate section before selecting a response.

Materials & experimental systems

n/a	Involved in the study
☒	☐ Antibodies
☒	☐ Eukaryotic cell lines
☒	☐ Palaeontology and archaeology
☒	☐ Animals and other organisms
☒	☐ Clinical data
☒	☐ Dual use research of concern
☒	☐ Plants

Methods

n/a	Involved in the study
☒	☐ ChIP-seq
☒	☐ Flow cytometry
☒	☐ MRI-based neuroimaging

Plants

Seed stocks	Since we only used public data, This item is not applicable to our study.
Novel plant genotypes	Since we only used public data, This item is not applicable to our study.
Authentication	Since we only used public data, This item is not applicable to our study.
